# Ventilation failure after lateral jackknife positioning for robot-assisted lung cancer surgery in a patient after lingula-sparing left upper lobectomy

**DOI:** 10.1186/s40981-018-0188-8

**Published:** 2018-06-21

**Authors:** Izumi Kawagoe, Masakazu Hayashida, Daizoh Satoh, Kenji Suzuki, Eiichi Inada

**Affiliations:** 1 Division of General Thoracic Anesthesia, Department of Anesthesiology and Pain Medicine, Juntendo University School of Medicine, Tokyo, Japan; 2 Division of Cardiovascular Anesthesia, Department of Anesthesiology and Pain Medicine, Juntendo University School of Medicine, Tokyo, Japan; 3 Division of Intensive Care Medicine, Department of Anesthesiology and Pain Medicine, Juntendo University School of Medicine, Tokyo, Japan; 4 Department of General Thoracic Surgery, Juntendo University School of Medicine, Tokyo, Japan; 5 Department of Anesthesiology and Pain Medicine, Juntendo University School of Medicine, Tokyo, Japan; 6 Tokyo, Japan

**Keywords:** Double-lumen tube, Lateral jackknife position, One-lung ventilation, Robot-assisted thoracic surgery, Silbroncho® tube

## Abstract

**Background:**

Ventilation failure commonly occurs when a standard left-sided double-lumen tube is used in patients after left upper lobectomy having remarkable angulation of the left main bronchus. We present a female without remarkable angulation, in whom ventilation failure occurred after lateral jackknife positioning.

**Case presentation:**

A 73-year-old female after lingula-sparing left upper lobectomy without remarkable angulation was scheduled for robot-assisted right upper lobectomy. Ventilation failure with a standard left-sided double-lumen tube occurred when she was placed not in the lateral position but in the lateral jackknife position required for robotic surgery. After replacement by the Silbroncho® left-sided double-lumen tube, adequate one-lung ventilation became possible.

**Conclusions:**

Ventilation failure with a standard tube may occur more easily when patients with bronchial angulation are placed in the lateral jackknife than lateral position due to posture-induced exacerbations of bronchial angulation. The Silbroncho® tube seems useful in such situations.

## Background

In patients undergoing second lung cancer surgery, special caution is required regarding bronchial displacement after previous surgery [1]. Particularly, patients after a left upper lobectomy commonly have U- or V-shaped angulation of the left main bronchus [2–5]. Although a standard left-sided double-lumen tube (DLT) is most commonly used to achieve one-lung ventilation (OLV), patients after a left upper lobectomy may encounter ventilation failure with this tube due to bronchial angulation [5]. We previously reported, in patients after this procedure, that ventilation failure could commonly occur in those having remarkable bronchial angulation, as defined by a combination of a wide angle (> 140°) between the trachea and left main bronchus (T-MB angle) and a narrow angle (< 100°) between the proximal and distal portions of the left main bronchus (PL-DL angle), whereas a standard left-sided DLT could be used safely in those without such remarkable angulation [5].

Recently, robot-assisted surgery is applied to lung cancer surgery because of its less invasiveness, better visualization of the surgical field, and easier accessibility to targets with more flexible devices, compared with open or video-assisted thoracic surgery [6]. However, the da Vinci surgical system demands a lateral jackknife position to maximize the intercostal spaces, thereby amplifying the room for the devices, and to prevent the pelvis from disturbing the movement of the large instrument arms [7, 8]. Therefore, special caution is required during robot-assisted thoracic surgery, regarding the lateral jackknife position [7, 8], in addition to cardiorespiratory effects of capnothorax during OLV [9].

Herein, we present a case of a female after a lingula-sparing left upper lobectomy scheduled for robot-assisted thoracic surgery, in whom ventilation failure with a standard left-sided DLT occurred after the lateral jackknife positioning. The patient provided written informed consent for, and the Institutional Review Board approved, publication of her case history (approval number, JHS 17-0051).

## Case presentation

**Fig. 1:**
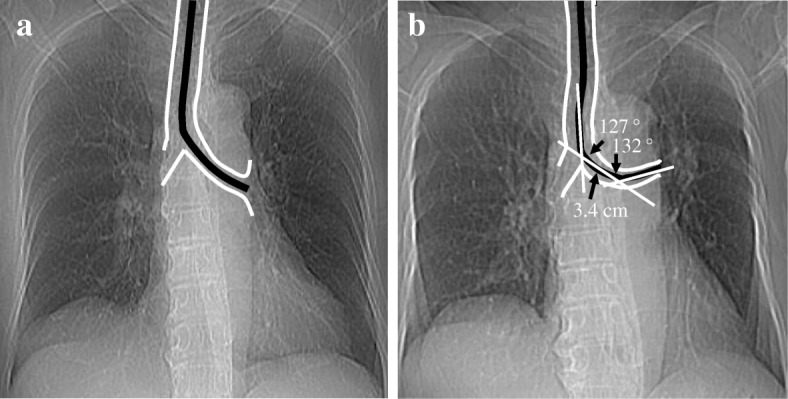
Chest X-rays before the previous lingula-sparing left upper lobectomy (**a**) and before the current robot-assisted right upper lobectomy (**b**). An outline and a centerline of the trachea and the left main bronchus are delineated with white and black lines, respectively, on each chest X-ray. The distance from the trachea to the angulation point is 3.4 cm. Note that compared with the chest X-ray **a**, the chest X-ray **b** exhibits mild angulation of the left main bronchus, indicated by a combination of the 127° T-MB angle and the 132° PL-DL angle, which does not meet the criteria for remarkable bronchial angulation, defined by a combination of a wide T-MB angle (> 140°) and a narrow PL-DL angle (< 100°) [6]. T-MB angle, angle formed by the trachea and left main bronchus; PL-DL angle, angle formed by the proximal and distal portions of the left main bronchus

A 73-year-old, 153-cm, 50-kg female was scheduled to undergo a robot-assisted right upper lobectomy for lung cancer. She had undergone a lingula-sparing left upper lobectomy 5 years before. A chest X-ray revealed angulation of the left main bronchus with a 3.4-cm distance from the trachea to the angulation point, 127° T-MB angle, and 132° PL-DL angle (Fig. 1), which did not meet the angle criteria for remarkable angulation. Therefore, a standard left-sided DLT was selected.

A thoracic epidural catheter was placed. After induction of general anesthesia with propofol, remifentanil, and rocuronium, a standard 35-Fr left-sided DLT (Portex Blue Line®, Smith Medical, Minneapolis, MN) was correctly placed with the video-assisted laryngoscope (McGrath MAC®, Medtronic, Minneapolis, MN) and a bronchoscope. The outlet of the bronchial port was partly faced with the bronchial wall. Test OLV with pressure-controlled ventilation with a peak pressure 20 cm H_2_O and positive end-expiratory pressure 4 cm H_2_O achieved a tidal volume above 250 mL while she was placed in the supine position. After placement in the left lateral position, test OLV could achieve a tidal volume above 200 mL. Although the tube outlet seemed almost fully faced with the bronchial wall, the bronchoscope could be advanced into the bronchus by flexing its tip. However, as soon as OLV was attempted after she was repositioned in the lateral jackknife position with the lowest chest raised and the lower trunk lowered by a flexed operating table [7], the tidal volume decreased to less than 50 mL. Adequate manual OLV was impossible. Bronchoscopically, the outlet was obstructed by the bronchial wall, and the bronchoscope could not be advanced beyond the tube outlet.

Immediately, the jackknife position was released, and adequate manual OLV became possible. The tube was exchanged for a 37-Fr left-sided DLT with a flexible wire-reinforced tip and a narrow bronchial cuff (Silbroncho®, Fuji Systems, Tokyo, Japan) [10], using an airway exchange catheter and the video-assisted laryngoscope while she remained in the lateral position. Further, under bronchoscopic guidance, the tube tip was placed below the angulation point and above the secondary carina so that the secondary carina was seen in front of the tube outlet. Thereafter, a tidal volume during OLV remained above 250 mL even after repositioning in the lateral jackknife position. The scheduled robotic surgery was completed within 146 min under combined general and epidural anesthesia. She was extubated 9 min after the surgery and discharged from the hospital on the fourth postoperative day.

## Discussion

In the female patient after a lingula-sparing left upper lobectomy scheduled for robot-assisted thoracic surgery, a standard left-sided DLT was selected because remarkable bronchial angulation was absent. Actually, OLV was possible while she was placed in the lateral position. However, it became impossible after the lateral jackknife positioning.

A lingula-sparing left upper lobectomy can be indicated for early-stage lung cancer in the left upper division [11]. Although no previous study has addressed bronchial angulation after this procedure, it would be less significant after this procedure than a left upper lobectomy, given the extents of lung resection. Actually, her bronchial angulation was not remarkable.

In the previous study in patients after a left upper lobectomy, ventilation failure with a standard left-sided DLT occurred in patients with remarkable angulation when they were placed not in the supine position but in the lateral position [5]. In the present patient without remarkable angulation, ventilation failure occurred when she was placed not in the lateral position but in the lateral jackknife position. The lateral position may exacerbate bronchial angulation because this position results in lung compression by the mediastinum and diaphragm [12]. Possibly, compression by the mediastinum exacerbates bronchial angulation, whereas compression by the diaphragm somewhat counteracts this exacerbation. The lateral jackknife position may exacerbate bronchial angulation further, because this position attenuates lung compression by the diaphragm due to a caudal shift of abdominal contents.

The present patient had a 3.4-cm distance from the trachea to the angulation point, suggesting that the tip of the 35-Fr Blue Line® DLT with a 3-cm bronchial port was located around the angulation point when the tube was correctly placed so that the endobronchial cuff was seen approximately 5 mm below the carina [12]. Therefore, the tube tip could be obstructed by the bronchial wall when the left main bronchus was bent more acutely due to the lateral jackknife position. Actually, the tube outlet was obstructed by the bronchial wall.

In such situations, a goal to prevent tube obstruction by placing the tube tip far above the angulation point [5] is achieved more easily with the Silbroncho® tube having a 2-cm bronchial port than standard left-sided DLTs having 3- or 3.5-cm bronchial ports [10], since the mean distance to the angulation point was 3.51 cm in the previous 18 patients after the lobectomy [5]. Likewise, a goal to prevent tube obstruction by placing the tube tip below the angulation point is achieved more easily with the Silbroncho® tube, since the flexible, wire-reinforced bronchial port allows it to follow the deformed bronchus without kinking [10], whereas inflexible, longer bronchial ports of standard left-sided DLTs may easily kink when they are involved in a posture-induced exacerbation of bronchial angulation. Because successful placement of the tube tip far above the angulation point may not always ensure successful OLV when exacerbated bronchial angulation causes severe bronchial kinking [3], we purposefully selected the larger 37-Fr Silbroncho® tube and placed its tip between the angulation point and secondary carina in an attempt to prevent tube obstruction by the bronchial wall, kinking of the tube, and even kinking of the bronchus.

Options alternative to this tube include a right-sided DLT [5], a bronchial blocker inserted through a single-lumen tube or DLT [5], and a single-lumen wire-reinforced tube placed in the left main bronchus [12]. However, the single-lumen tube is cumbersome to use because its withdrawal and advancement under bronchoscopic guidance must be repeated during inflation-deflation cycles repeated for the assessment of surgical air leaks**.** Right-sided DLTs are more difficult to position for adequate lung isolation and ventilation of the right upper lobe, compared with left-sided DLTs [12, 13]. With a bronchial blocker, deflation of the isolated lung is slower and suctioning beyond the blocker is less effective, compared with DLTs [13]. An endobronchial cuff of a right-sided DLT or bronchial blocker is easier to dislodge during surgical manipulation of the right lung, compared with left-sided DLTs [13]. Therefore, the Silbroncho® left-sided DLT seemed a suitable option, although bronchoscopic guidance is more frequently required for its correct placement, e.g., by preventing intra-tracheal kinking of its flexible bronchial port, compared with standard DLTs [14].

In a female after a lingula-sparing left upper lobectomy without remarkable bronchial angulation, ventilation failure with a standard left-sided DLT developed after she was placed in not the lateral but lateral jackknife position. Eventually, successful OLV could be achieved with the Silbroncho® left-sided DLT. Our experience suggests that ventilation failure with standard left-sided DLTs can develop more easily when patients with left bronchial angulation are placed in the lateral jackknife position required for robotic surgery than in the lateral position used for more conventional thoracic surgery. The Silbroncho® left-sided DLT is useful to overcome such problems.

## Abbreviations

DLT: Double-lumen tube
OLV: One-lung ventilation
PL-DL angle: Angle formed by the proximal and distal portions of the left main bronchus
T-MB angle: Angle formed by the trachea and left main bronchus
